# On-going malaria transmission in The Gambia despite high coverage of control interventions: a nationwide cross-sectional survey

**DOI:** 10.1186/s12936-015-0829-6

**Published:** 2015-08-14

**Authors:** Julia Mwesigwa, Joseph Okebe, Muna Affara, Gian Luca Di Tanna, Davis Nwakanma, Omar Janha, Kevin Opondo, Koen Peeters Grietens, Jane Achan, Umberto D’Alessandro

**Affiliations:** Medical Research Council Unit, PO Box 273, Banjul, The Gambia; London School of Hygiene and Tropical Medicine, London, UK; Institute of Tropical Medicine, Antwerp, Belgium; School of International Health Development, Nagasaki University, Nagasaki, Japan; Partners for Applied Social Sciences (PASS) International, Tessenderlo, Belgium

**Keywords:** Malaria transmission, Asymptomatic parasitaemia, Heterogeneity, The Gambia

## Abstract

**Background:**

As indicators of burden of malaria have substantially decreased in The Gambia, reaching a pre-elimination status may be attainable. Achieving this goal requires in-depth understanding of the current burden of *Plasmodium falciparum* infection.

**Methods:**

A nationwide cross-sectional survey was conducted in 2012 to determine the prevalence of *P.falciparum* infection, and to describe its heterogeneity and associated risk factors. Finger-prick blood samples were collected for microscopy, species-specific PCR and haemoglobin measurement.

**Results:**

A total of 9,094 participants were included and median age was 11.9 years (IQR 5, 28). Overall prevalence of *P. falciparum* was 16.01 % with marked heterogeneity between sites (4.32–36.75 %) and within villages in each site (1.63–49.13 %). Across all sites, 51.17 % (745/1,456) of infections were asymptomatic and 35.61 % (448/1,258) were sub-microscopic. The odds of *P. falciparum* infection were higher in older children; 5–15 years (OR = 1.90; 95 % CI 1.60–2.26), adults (OR = 1.48; 95 % CI 1.24–1.78) and participants with moderate anaemia (OR = 1.62; 95 % CI 1.32–1.99).

**Conclusions:**

The current malaria control interventions are not sufficient to interrupt transmission in The Gambia as malaria prevalence is still relatively high in the eastern part of the country. New interventions aiming at interrupting transmission are needed and should be urgently evaluated.

## Background

The last decade has witnessed major progress in the fight against malaria as the burden has substantially reduced, even in sub-Saharan Africa, although malaria still contributes significantly to both morbidity and mortality [1, 2]. The Gambia is one of the African countries where a substantial decline has been observed [3, 4]. This decline resulted from the scaling-up of malaria control intervention [5], which includes: increased availability and access to long-lasting insecticide bed nets (LLINs), integrated vector control interventions such as indoor residual spraying (IRS), strengthened case management with rapid diagnostic tests (RDTs), and artemisinin combination therapy (ACT). Despite the scaling-up of these interventions, malaria transmission, which is highly seasonal, has not been interrupted. Earlier studies in schoolchildren [6, 7] showed marked heterogeneity in malaria prevalence across the country and significant seasonal variation [8]. In addition, high resistance to DDT and pyrethroids observed in some areas raises concerns about the impact of current vector control interventions [9, 10].

As the country aims for pre-elimination status, gaining a detailed understanding of the current distribution of malaria infection, characterizing its heterogeneity across the country and further identifying asymptomatic carriers is critical as these are important elements for disease surveillance and targeting control efforts. Whereas health facility data have previously been used to describe malaria morbidity trends [3], this information is greatly limited by incompleteness, differing malaria case definitions and the lack of data on asymptomatic carrier status. This cross-sectional survey was, therefore, conducted to better characterize the dynamics of malaria transmission in The Gambia and to identify the determinants of its heterogeneity.

## Methods

**Fig. 1:**
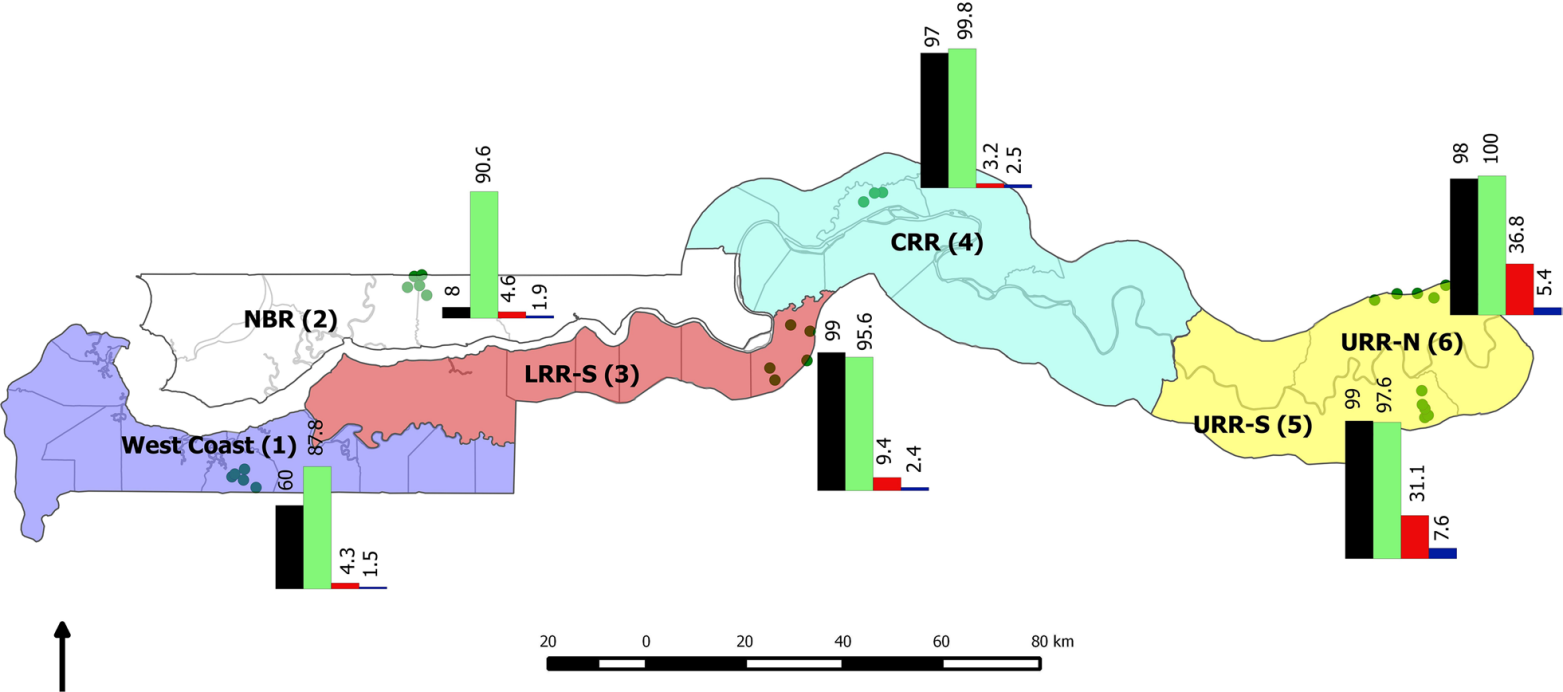
Study sites location, long-lasting insecticidal net coverage, indoor residual spraying coverage, and severe anaemia and *Plasmodium falciparum* prevalence by study sites. *Black* IRS. *Green* LLINs. *Red* Plasmodium falciparum prevalence. *Blue* Severe anaemia.

The Gambia is divided into five administrative regions, namely West Coast (WC), North bank region (NBR), Lower River (LRR), Central River (CRR) and Upper River (URR) regions (Fig. 1). It is characterized by a long dry season from mid-October to mid-June followed by a single short rainy season from June to September when the average daily temperature is at 28 °C, creating a favourable environment for *Anopheles gambiae* mosquitoes. Malaria transmission occurs almost exclusively during the rainy season and immediately afterwards, until December-January. The entomological inoculation rate varies from 0 to 166 infective bites per person per year [11]. For this study, the country’s five geographical regions were used; WC, NBR, LRR- south bank, CRR- north bank and subdivided the URR into the north and south bank. In each of these regions, one primary school with the highest seroprevalence of anti-malarial antibodies (MSP1_19_) among school children was identified using findings from a previous 2012 dry season nationwide seroprevalence survey [7]. In November 2012, six neighbouring villages around each of these schools, with populations between 100 and 500 were selected for inclusion in this survey. In villages with less than 300 inhabitants, the whole population was included while in larger villages the first 350 participants were consecutively enrolled. In all the villages participants were requested to gather at a central location for the survey, usually the village central meeting area. Infants less than 6 months of age or individuals who had stayed less than 4 weeks in the village were excluded. Village locations were mapped using a hand-held global positioning system (GPS) (Garmin eTrex^®^ 10).

Each participant was allocated a unique study ID code and individual interviews were conducted for older children (12–17 years) and adults, while parents/guardians completed the questionnaire for younger children (6 months–11 years). Individual participant information on demographic and general health indicators such as history of fever, sleeping habits (including outdoor or out in the field) and travel outside the village in the past month and IRS were collected using a structured questionnaire. Individual participants were asked if their house had received IRS in the last 1 year and in addition if they had slept under a LLIN the previous night. Axillary temperature was measured using a digital thermometer. A finger-prick blood sample was collected from each participant on a blood slide for microscopy and spotted on filter paper (Whatman 3 Corporation, Florham Park, NJ, USA) for molecular analysis. Haemoglobin (Hb) was measured with the HaemoCue 301 machine (Ängelholm, Sweden) following the manufacturer’s instructions.

### Laboratory procedures

To determine parasite density, microscopy was read on all samples that tested positive by PCR. Slides were stained with 2.5 % buffered Giemsa (pH 7.2) for 30 min and double read by trained microscopists who were blinded to the survey data of the participants. Slide smears were declared negative if no parasite was seen after examining 200 high power fields (HPF). If positive, parasites were counted against 500 white blood cells (WBCs and parasite densities were calculated assuming 8,000 WBC per μl of blood. A 20 % error check was used to identify discrepancies between slide readers. All discordant results were read by a senior microscopist who was also blinded to participant survey data and these results were taken as the final read.

For diagnostic PCR, three 6-mm dried filter-paper blood spots (DBS) were punched into a 96-well plate. DNA extraction was carried out using the automated QIAxtractor robot (Qiagen). Negative and positive (3D7) controls were included to control for cross contamination and DNA extraction efficiency, respectively. The DBS were lysed by incubating them in tissue digest buffer at 60 °C for 1 h and digested eluates were applied onto capture plates, washed, and the DNA eluted into 80 µl. The extracted DNA (4 µl) was used in a nested PCR, amplifying the multicopy *Plasmodium* ribosomal RNA gene sequences using genus and species specific primers [12]. All PCR products were run using the QIAxcel capillary electrophoresis system (Qiagen), using the screening cartridge and 15-1,000 bp-alignment marker. Results were exported and double scored using both the QIAxcel binary scoring function and manually by visualization of the gel images and discrepancies were scored by a third independent reader. All readers were blinded to participant survey data.

### Statistics

The sample size was based on the desired precision of the prevalence of infection by village; 300 individuals allowed an estimation of the prevalence with the following precision: 1 % (95 % CI 0.2–3 %); 5 % (3–8 %); 12 % (8–16 %), and 20 % (16–25 %). Data from questionnaires were verified for completeness and all data were double entered and verified using Microsoft Access database (Microsoft Corp., Redmond, USA). Errors in data entry were detected by running consistency checks before exportation to STATA 13.0 (StataCorp, College Station, Texas, USA) for analysis. Age groups were categorized into <5 years, ≥5 to ≤15 years and >15 years [13, 14]; anaemia was defined according to the WHO Haemoglobin concentration for diagnosis of anaemia and assessment of severity; as severe (<8.0 g/dl), moderate (8.0–10.9 g/dl), or mild (11.0–12.9 g/dl) and no anaemia (≥12.9 g/dl) [15]. Carriage of *Plasmodium falciparum* infection was defined as parasitaemia detected by PCR at screening [16]. LLIN coverage was reported as the proportion of the population that slept under an LLIN the previous night.

Descriptive statistics are presented for continuous variables (median and interquartile range), and proportions for categorical variables and all point estimates presented with 95 % confidence intervals calculated using the Wilson score method with continuity correction. Uni- and multi-variable logistic regression analyses were performed to determine the independent predictors of *P. falciparum* infection. To account for missing data, complete case analyses were presented along with multiple imputation models (50 iterations), which allowed for the inclusion of partially observed cases. For multiple imputation analysis, a fully conditional model specification [17, 18] was adopted in which a logistic model was used for binary variables (gender, slept under a LLIN the previous night, sleep outdoors at night, IRS, sleep in the fields, and used artemether-lumefantrine the previous week) and an ordinal logistic model was performed for age and anaemia categorical variables. There was an allowance for clustering between and within the six sites in the uni-multivariable models. For age and anaemia, categorical variables, tests for trend and significance of the associations were performed in the multivariable models and, a goodness of fit test performed for the final model. For the analysis of microscopy, the median asexual and sexual parasite densities were calculated and the proportions of parasitaemia determined. The geolocations and *P. falciparum* prevalence data of each village were imported from Microsoft access and transferred separately onto a layer containing the base map of The Gambia generated using Quantum GIS 2.0.1 (QGIS 2.0.1). The village GPS points with the corresponding variable attributes were then displayed on the new layer and the maps were exported as jpeg, 800dpi file format (Fig. 1).

This study was approved by the Gambia Government/MRC Joint Ethics Committee (SCC1318). Verbal consent for participation was first obtained during the village sensitization meetings. Written informed consent was obtained for all participants, with parents/guardians providing written consent for children less than 12 years. Assent was also obtained from children aged between 12 and 17 years.

## Results

Over 3 weeks in November 2012, a total of 10,191 participants were enrolled from 36 villages. Of these, 9,094 (89.24 %) participants with PCR results and complete questionnaire data were included in this analysis. The median age was 11.9 years (IQR 5, 28) and the majority [59.57 % (5,417/9,094); 95 % CI 58.55–60.58 %] were females (Table 1). The mean (SD) haemoglobin was 12.7 g/dl (2.26) and the overall prevalence of severe anaemia was [3.77 % (339/8,991); 95 % CI 3.39–4.19 %]. The highest prevalence of severe anaemia was among children less than 5 years [7.52 % (184/2,448); 95 % CI 6.52–8.65 %] and also in the eastern part of the country (Table 2).

**Table 1:** Demographic and clinical profiles of the study population

Variable	N (%)
Age (N = 9,079)	
<5 years	2,477 (27.28)
5–15 years	2,811 (30.96)
>15 years	3,791 (41.76)
Gender (N = 9,094)	
Female	5,417 (59.57)
Male	3,677 (40.43)
Anaemia (N = 8,991)	
No anaemia (>12.9 g/dl)	2,198 (24.45)
Mild (11.0–12.9 g/dl)	3,783 (42.08)
Moderate (8.0–10.9 g/dl)	2,671 (29.70)
Severe (<8.0 g/dl)	339 (3.77)
Has received indoor residual spraying in past 1 year (N = 9,094)	
Yes	7,170 (78.84)
No	1,924 (21.16)
Has slept under a LLIN the previous night (N = 8,621)	
Yes	8,244 (95.63)
No	377 (4.37)
Has slept outdoors at night (N = 9,064)	
Yes	3,807 (42.0)
No	5,257 (58.0)
Has used an LLIN outdoors at night (N = 3,689)	
Yes	1,405 (38.09)
No	2,284 (61.91)
Has history of fever in the last 24 h (N = 9,094)	
Yes	4,195 (46.13)
No	4,899 (53.87)
Presence of fever (axillary temperature ≥37.5 °C) (N = 9,094)	
Yes	498 (5.48)
No	8,596 (94.52)
Has taken artemether-lumefantrine in past 2 weeks (N = 9,094)	
Yes	174 (1.91)
No	8,920 (98.09)

**Table 2:** *Plasmodium falciparum* prevalence and risk factors by site (%)

	West Coast	North bank region	LRR-south bank	CRR-north bank	URR-south bank	URR-north bank
*Plasmodium falciparum* prevalence	53/1,227 (4.32)	65/1,417 (4.59)	127/1,357 (9.36)	53/1,676 (3.16)	538/1,730 (31.10)	620/1,687 (36.75)
Age, median (IQR)	15 (5, 36)	11 (5, 25)	12 (5, 30)	10 (5, 27)	12 (5, 30)	11 (5, 24)
Anaemia	N = 1,226	N = 1,329	N = 1,357	N = 1,674	N = 1,721	N = 1,684
No anaemia	505 (41.19)	377 (28.37)	360 (26.53)	458 (27.36)	215 (12.49)	283 (16.81)
Mild	531 (43.31)	583 (43.87)	590 (43.48)	701 (41.88)	662 (38.47)	716 (42.52)
Moderate	172 (14.03)	344 (25.88)	374 (27.56)	473 (28.26)	714 (41.49)	594 (35.27)
Severe	18 (1.47)	25 (1.88)	33 (2.43)	42 (2.51)	130 (7.55)	91 (5.40)
Has slept under a LLIN last night	1,075/1,225 (87.76)	1,112/1,227 (90.63)	1,266/1,325 (95.55)	1,614/1,618 (99.75)	1,616/1,653 (97.76)	1,561/1,573 (99.24)
Has slept outdoors	156/1,216 (12.83)	430/1,415 (30.39)	405/1,350 (30.00)	976/1,675 (58.27)	769/1,724 (44.61)	1,071/1,684 (63.60)
Indoor residual spraying	744/1,225 (60.73)	110/1,417 (7.76)	1,319/1,335 (98.80)	1,620/1,673 (96.83)	1,719/1,722 (99.83)	1,658/1,681 (98.63)

The proportion of participants that reported sleeping under an LLIN the previous night was high [95.63 % (8,244/8,621); 95 % CI 95.17–96.04 %] across all sites while [78.84 % (7,170/9,094); 95 % CI 77.99–79.68 %] of the participants reported having received IRS in their households the previous year. A relatively high proportion of individuals [42.0 % (3,807/9,064); 95 % CI 40.98–43.03 %] slept out doors at night; this was more frequent in the eastern part of the country, i.e., URR-north bank [63.60 % (1,071/1,684); 95 % CI 61.24–65.89 %], CRR-north bank [58.27 % (976/1,675); 95 % CI 55.86–60.64 %] and URR-south bank [44.61 % (769/1,724); 95 % CI 42.24–46.99 %] (Table 2). Few individuals [3.19 % (291/9,094); 95 % CI 2.85–3.59 %] reported travelling outside their village or sleeping in the fields during this period. Although [46.13 % (4,195/9,094) 95 % CI 45.10–47.16 %] of the participants reported a history of fever in the preceding 24 h at the time of the survey, only [5.48 % (498/9,094); 95 % CI 5.02–5.97 %] had a documented fever (axillary temperature ≥37.5 °C). Only 1.9 % of participants [(174/9,094); 95 % CI 1.65–2.22 %] reported having taken artemether-lumefantrine, the first-line anti-malarial treatment at the time of the survey (Table 1). Of the 865 slides read by microscopy, the median (IQR) asexual *P. falciparum* density was 2,000 µl (192, 12,720) and the median (IQR) gametocyte density (N = 225) was 80 µl (32,224).

The overall prevalence of *P. falciparum* infection as determined by PCR was [16.01 % (1,456/9,094); 95 % CI 15.27–16.78 %] with significant heterogeneity observed between sites and within villages in each site. *Plasmodium falciparum* prevalence was highest in the eastern part of the country, with average parasite prevalence in the URR-south bank of [31.09 % (538/1,730); 95 % CI 28.93–33.35 %], ranging from [18.15 % (49/270): 95 % CI 13.84–23.38 %] in Waliba Kunda to [49.13 % (169/344); 95 % CI 34.45–39.11 %] in Madina Samako, and [36.75 % (620/1,687); 95 % CI 34.45–39.11 %] in the north bank, ranging from [29.86 % (103/345); 95 % CI 25.13–35.03 %] in Maka Masireh to [47.42 % (92/194); 95 % CI 40.26–54.69 %] in Jecka (Table 3). Conversely, in the other sites, the average prevalence was below 5 %, with the exception of LRR-south bank where it was [9.36 % (127/1,357); 95 % CI 7.89–11.06 %]. Notably, there was statistically significant heterogeneity (P ≤ 0.001) observed within these low prevalence sites with some villages having relatively high prevalence like Ndemban Tenda in WC with a prevalence of [7.86 % (11/140); 95 % CI 4.18–13.95 %] and Wellingara Chogen in NBR with [10.24 % (21/205); 95 % CI 6.60–15.43 %] (Table 3).

**Table 3:** Prevalence of *Plasmodium falciparum* as determined by PCR by site and village

Site	Village	*Plasmodium falciparum* prevalence
One	Bessi	5/307 (1.63)
WC	Jagil	4/135 (2.96)
	Ndemban Jola	11/269 (4.09)
	Ndemban	14/272 (5.15)
	Kanjanbina	8/104 (7.69)
	Ndemban Tenda	11/140 (7.86)
	Sub-total	53/1,227 (4.32)
Two	Yallal Ba	5/255 (1.96)
NBR	Pallen Hamdalia	8/305 (2.62)
	Daru Rilwan	6/243 (2.47)
	Mbamori Kunda	8/184 (4.35)
	Tallen Fula	17/225 (7.56)
	Wellingara Chogen	21/205 (10.24)
	Sub-total	65/1,417 (4.59)
Three	Sinchu Njengudi	5/100 (5.00)
LRR-south bank	Darsilame	21/308 (6.82)
	Nyawurulung	17/208 (8.17)
	Baro Kunda	22/266 (8.27)
	Jassong	17/176 (9.66)
	Dongoro Ba	45/299 (15.05)
	Sub-total	127/1,357 (9.36)
Four	Sinchu Tamsfir	2/348 (0.57)
CRR-north bank	Ngedden	3/179 (1.68)
	Buduk	11/302 (3.64)
	Sare Janko	13/349 (3.72)
	Mbaien Burama	7/177(3.95)
	Sare Seedy	17/321 (5.30)
	Sub-total	53/1,676 (3.16)
Five	Waliba Kunda	49/270 (18.15)
URR-south bank	Njayel	65/307 (21.17)
	Dingiri	90/344 (26.16)
	Sare Gela	94/267 (35.21)
	Sendebu	71/198 (35.86)
	Madina Samako	169/344 (49.13)
	Sub-total	538/1,730 (31.09)
Six	Maka Masireh	103/345 (29.86)
URR-north bank	Sare Wuro	68/246 (27.64)
	Wellingara Yarel	116/351 (33.05)
	Gunjur Koto	86/203 (42.36)
	Mure Kunda	155/348 (44.54)
	Jecka	92/194 (47.42)
	Sub-total	620/1,687 (36.75)

**Fig. 2:**
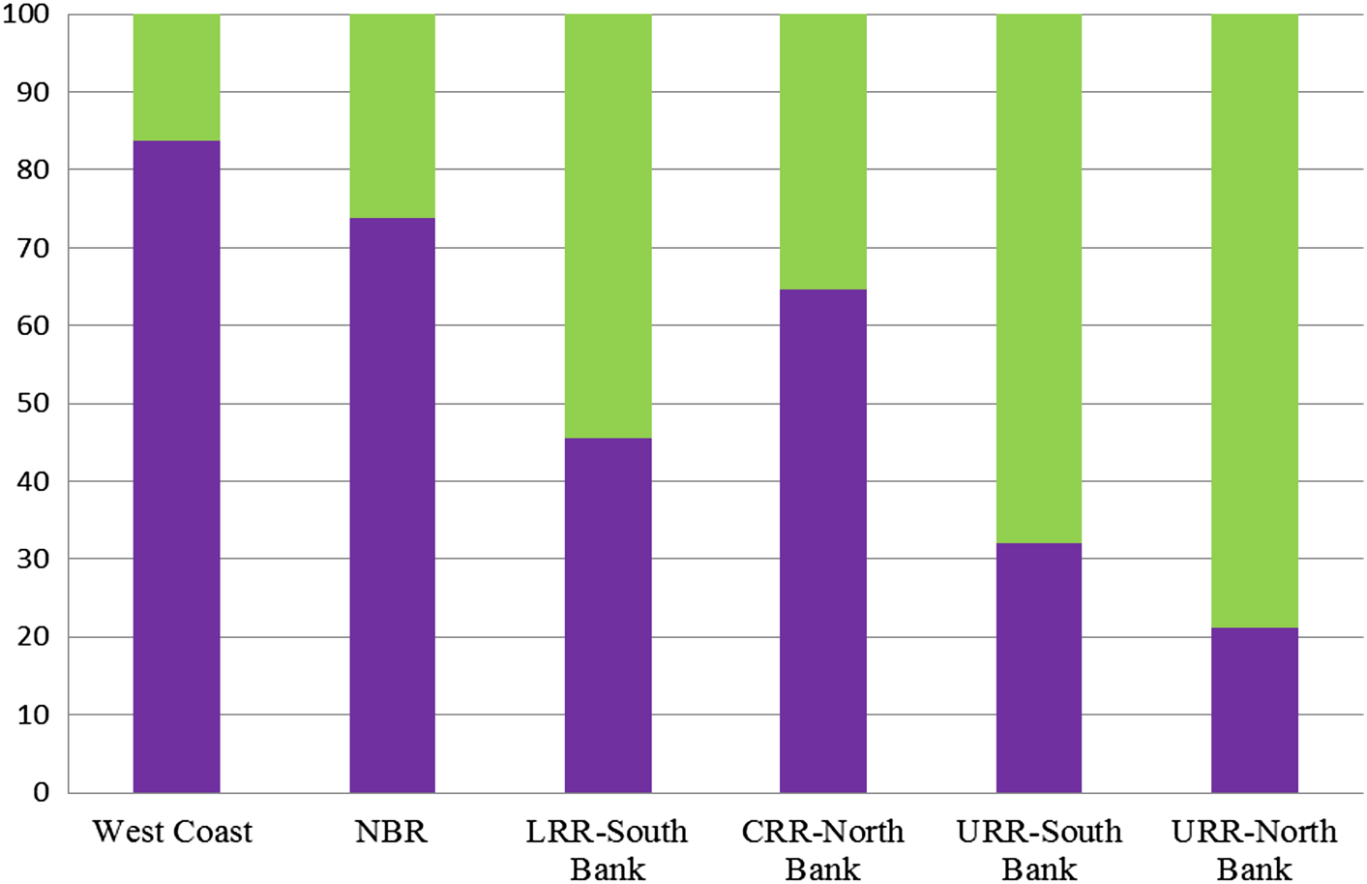
Proportions of sub-microscopic parasitaemia across the regions. *Green* Proportion of microcopy asexual parasitaemia and PCR. *Purple* Proportion of sub-microscopic parasitaemia.

Among the participants with *P. falciparum* infection, [51.17 % (745/1,456); 95 % CI 48.57–53.76 %] across all sites were asymptomatic, i.e., without fever or history of fever. The relative distribution of asymptomatic *versus* symptomatic varied significantly by region. The proportion of asymptomatic cases ranged from [25.20 % (31/123); 95 % CI 18.01–33.98 %] in the LRR-south bank and URR-south bank to [78.39 % (486/620); 95 % CI 74.89–81.52 %] in URR-north bank and [92.31 % (60/65); 95 % CI 82.25–97.13 %] in NBR. Conversely, the highest proportion of individuals positive by PCR that had fever or history of fever was in the URR-south bank [75.84 % (408/538); 95 % CI 71.95–79.35 %] and in the CRR-north bank [73.23 % (93/127); 95 % CI 64.51–80.51 %]. Overall, only [14.35 % (209/1,456); 95 % CI 12.61–16.29 %] of participants with *P. falciparum* infection across the six sites had documented fever. The overall prevalence of sub-microscopic parasitaemia was [35.61 % (448/1,258); 95 % CI 32.97–38.34 %] with an inverse relationship between the prevalence of *P. falciparum* parasitaemia and distribution of sub-microscopic parasitaemia (Fig. 2). The regions with the highest parasite prevalence in the URR had the lowest proportions of sub-microscopic parasitaemia; URR- south bank [32.10 % (174/542); 95 % CI 28.22–36.24 %] and [21.27 % (87/409); 95 % CI 17.47–25.62 %] in URR-north bank. The areas with low overall parasite prevalence had significantly higher proportions of sub-microscopic parasitaemia ranging from [64.71 % (33/51); 95 % CI 50.0–77.20 %] in CRR-north bank to [83.72 % (36/43); 95 % CI 68.70–92.67 %] in WC. Among participants with gametocytaemia, [21.78 % (44/202); 95 % CI 16.43–28.24 %] were at sub-microscopic densities.

In summary, results from the univariable analysis with clustering within and between sites showed higher odds of *P.falciparum* infection among older children (OR = 1.55; 95 % CI 1.32–1.82) compared to children <5 years, participants with moderate (OR = 1.38; 95 % CI 1.15–1.65) and severe anaemia (OR = 2.44; 95 % CI 1.84–3.25) compared to those with no anaemia whereas, sleeping under a LLIN the previous night was protective against *P. falciparum* infection (OR = 0.58; 95 % CI 0.41–0.81) and women had lower odds of infection compared to males (OR = 0.86; 95 % CI 0.76–0.98). In the multivariable analyses, with clustering with-in and between sites the odds of *P. falciparum* infection were significantly higher among older children (OR = 1.90; 95 % CI 1.60–2.26; P ≤ 0.001) and adults (≥15 years) (OR = 1.48; 95 % CI 1.24–1.78; P ≤ 0.001) compared to younger children (<5 years). In addition, females had a significantly lower odds of parasitaemia (OR = 0.87; 95 % CI 0.76–1.00; P = 0.05) compared to males. *Plasmodium falciparum* infection was also strongly associated with anaemia, with the odds of parasitaemia increasing with severity of anaemia. The odds of infection were three-fold higher among participants with severe anaemia (moderate: OR = 1.62; 95 % CI 1.32–1.99; P ≤ 0.001; severe: OR = 3.11; 95 % CI 2.27–4.26; P ≤ 0.001) compared to participants with no anaemia (test for trend anaemia P = 0.002). Sleeping under a LLIN the previous night was protective against malaria infection (OR = 0.58; 95 % CI 0.41–0.82; P ≤ 0.001). In NBR, sleeping outdoors at night was associated with a higher odds of *P. falciparum* infection (OR = 1.98, 95 % CI 1.16–3.41; P = 0.01). However, the association was not statistically significant in the overall uni-multivariable analyses. “Sleeping outdoors at night”, “indoor residual spraying” and “sleeping out in the fields” were not predictors of *P.falciparum* infection. There were no differences in the covariates predicting malaria infection when compared to the multilevel, multivariable, parsimonious model, suggesting missing data did not affect the observed trends in association (Table 4).

**Table 4:** Risk factors for *Plasmodium falciparum* infection (uni- and multi-variable analysis, complete case and multiple imputation analyses, multilevel logistic model)

	Univariable analysis		Multivariable analysis (full model, complete case analysis) (n = 8,362)		Multivariable analysis (full model, multiple imputation analysis) (n = 9,094, 50 imputations)	
	OR (95 % CI)	P value	OR (95 % CI)	P value	OR (95 % CI)	P value
Age						
≤5 years (N = 2,477)	1		1		1	
5–14.9 years (N = 2,811)	1.55 (1.32–1.82)	<0.001	1.90 (1.60–2.26)	<0.001	1.84 (1.56–2.18)	<0.001
≥15 years (N = 3,791)	1.10 (0.94–1.29)	0.24	1.48 (1.24–1.78)	<0.001	1.43 (1.20–1.71)	<0.001
Gender						
Male (N = 5,417)	1		1		1	
Female (N = 3,670)	0.86 (0.76–0.98)	0.02	0.87 (0.76–1.00)	0.05	0.85 (0.75–0.97)	0.01
Anaemia						
No anaemia (N = 2,198)	1		1		1	
Mild (N = 3,783)	1.02 (0.85–1.22)	0.85	1.08 (0.89–1.32)	0.42	1.08 (0.89–1.30)	0.44
Moderate (N = 2,671)	1.38 (1.15–1.65)	<0.001	1.62 (1.32–1.99)	<0.001	1.62 (1.33–1.97)	<0.001
Severe (N = 137)	2.44 (1.84–3.25)	<0.001	3.11(2.27–4.26)	<0.001	3.05 (2.25–4.13)	<0.001
Has slept under a LLIN last night						
No (N = 377)	1		1		1	
Yes (N = 8,244)	0.58 (0.41–0.81)	<0.001	0.58 (0.41–0.82)	0.003	0.61 (0.43–0.86)	0.005
Has slept out doors						
No (N = 5,257)	1		1		1	
Yes (N = 3,807)	1.04 (0.92–1.19)	0.51	1.01(0.88–1.16)	0.87	1.04 (0.91–1.18)	0.56
Indoor residual spraying						
No (N = 1,883)	1					
Yes (N = 7,170)	0.86 (0.59–1.27)	0.46	0.86 (0.57–1.29)	0.45	0.92 (0.63–1.36)	0.69
Has slept out in the fields						
No (N = 8,727)	1		1		1	
Yes (N = 321)	0.78 (0.51–1.19)	0.26	0.81 (0.51–1.28)	0.37	0.83 (0.54–1.27)	0.38
Used Artemether-lumefantrine in past week						
No (N = 8,881)	1		1		1	
Yes (N = 174)	0.78 (0.51–1.20)	0.98	0.77 (0.49–1.20)	0.25	0.75 (0.48–1.15)	0.19

## Discussion

Whereas the burden of malaria in The Gambia has decreased significantly over the last 10 years [3, 4], probably because of the moderately high coverage of preventive interventions (LLINs and IRS) and free access to ACT, malaria transmission is ongoing. The prevalence of *P. falciparum* infection in villages in the NBR (site 2) was similar to that reported from community-based surveys in 2009 [8], implying that despite a substantial reduction, malaria transmission is still ongoing. The observed prevalence of infection close to 50 % in some villages in the eastern part of the country is comparable to the prevalence reported from high transmission areas in Tanzania [13], Gabon [19] or Burkina Faso [20]. In the rest of the country, prevalence was much lower and heterogeneous, with a five- to eight-fold difference between neighbouring villages. Although the selection of villages for this survey was done according to documented high prevalence of anti-malarial antibodies in school children, the results reported are likely to be a fair representative of the malaria transmission in The Gambia as geographical representation was taken into account.

The difference in transmission between the eastern part and the rest of the country cannot be explained by different coverage of LLINs, as their reported use is relatively high throughout the country and LLIN use reduced odds infection of *P. falciparum* infection. However, the high resistance to DDT and pyrethroids observed in eastern Gambia [9, 10] and in Senegal [21] could be responsible for the higher malaria prevalence. Other factors to consider are increased outdoor biting of the local vectors in response to environmental changes as has been reported previously [22, 23], the differences in vector composition across the regions could also contribute to the higher prevalence of *P. falciparum* in the eastern region. *A. gambiae* s.s and *A. arabiensis* are the predominant and in general most efficient vectors in the east, while other areas of the country have less efficient vectors, such as *A. melas* and *A. coluzzii*. In addition, this region has more difficult access to health care and prompt treatment. For the latter, the eastern part of The Gambia, where the highest malaria prevalence has been observed, is the least developed and the farthest from the capital.

The low malaria prevalence observed in the western part of the country is consistent with earlier reports in 2008 [8] indicating that 4 years later and despite high coverage of preventive interventions, the human reservoir of infection has not decreased substantially. A significant proportion of infections in this region were also sub-microscopic, implying that they would be missed by routinely used diagnostic tools and would therefore go untreated, hence contributing to maintain the human reservoir of infection and transmission. Conversely in the URR-south bank and NBR, whereas the majority of infections were detected by microscopy, a significant proportion of the participants were asymptomatic and thus unlikely to attend health facilities. Such individuals could therefore remain parasitaemic in the community and maintain residual transmission as well.

Identifying individuals who constitute this reservoir of infection would be extremely important for the identification of ‘hot spots’ in these villages, and designing and targeting interventions aimed at further decreasing malaria transmission. Assuming such carriers are mostly the same individuals from one transmission season to the other, because of environmental factors, such as higher exposure to infectious bites, residing in households near breeding sites, or genetic factors [24], targeting them for treatment could interrupt transmission. An intense follow-up of some villages included in this survey and covering several transmission seasons is currently ongoing and should be able to prove or disprove this hypothesis.

As previously reported [25, 26], about 36 % of PCR-positive samples were negative by microscopy and thus had sub-microscopic infections. The role of such infections in maintaining transmission remains unclear and urgently needs to be addressed [27]. Sub-microscopic infections may be important in maintaining transmission; an increasing body of evidence indicates that a large proportion of gametocyte carriers remain undetected by microscopy, and gametocytes may actually be present in the majority of infections, albeit at low densities [28]. When considering that the association between gametocyte density and mosquito infection rate is not very strong and is most variable at low gametocyte concentrations [28], the contribution of the carriers identified within this survey to the maintenance of malaria transmission is plausible. The challenge is to identify and treat them in the most efficient way. Active case detection strategies are increasingly popular across a number of countries although there is little evidence of their effectiveness [29]. Their main limitation is the sensitivity of the diagnostic screening tools used, which would miss an important proportion of sub-microscopic carriers unless field-adapted molecular diagnostic methods are used [30, 31]. Other chemoprevention approaches, such as seasonal chemoprevention in children less than 5 years of age recently implemented in some regions of The Gambia and mass drug administration aimed at eliminating the reservoir of infection should be considered as additional interventions, especially in areas where transmission has not declined significantly.

The prevalence of asymptomatic parasitaemia was significantly higher in the older children and adults, possibly the result of stronger immunity, making them able to tolerate [32] but not eliminate a malaria infection. Therefore, infections acquired among older children and adults during the current transmission season may remain asymptomatic and untreated, resulting in the higher prevalence observed in these age groups. Nevertheless, such infections are not without consequences as infected individuals had a significantly higher risk of anaemia. This suggests that even if not associated with acute symptoms, malaria infections do have a deleterious effect on the health of infected individuals. A similar association was described in Ghana [33], Cameroon [34] and Tanzania [13] but not among Ugandan schoolchildren [35].

## Conclusion

When considering the distribution of malaria infection across The Gambia, two main strata can be identified: the eastern part where transmission is still relatively intense, with villages in which a third to a half of the population infected, and the western part with low prevalence. Within each stratum, there is considerable heterogeneity between villages. It is remarkable to notice that even in the low endemicity stratum and despite high coverage of preventive and curative interventions, transmission has been maintained over the years. Obviously, current interventions are not sufficient to interrupt transmission in the current context of modified human and vector behaviour and new approaches need to be urgently evaluated. The low endemicity stratum in The Gambia offers an ideal setting to test new interventions aiming at interrupting malaria transmission.
